# Hypercortisolaemia due to ectopic adrenocorticotropic hormone secretion by a nasal paraganglioma: a case report and review of the literature

**DOI:** 10.1186/1756-0500-6-331

**Published:** 2013-08-19

**Authors:** Theodoros Thomas, Steffen Zender, Christoph Terkamp, Elmar Jaeckel, Michael P Manns

**Affiliations:** 1 Department of Gastroenterology, Hepatology and Endocrinology, Hannover Medical School, Carl-Neuberg Str. 1, 30625, Hannover, Germany

**Keywords:** Cushing, Paraganglioma, Nasal, ACTH, Ectopic

## Abstract

**Background:**

Adrenocorticotropic hormone-producing extraadrenal paragangliomas are extremely rare. We present a case of severe hypercortisolemia due to ectopic adrenocorticotropic hormone secretion by a nasal paraganglioma.

**Case presentation:**

A 70-year-old Caucasian woman was emergently admitted to our department with supraventricular tachycardia, oedema of face and extremities and hypertensive crisis. Initial laboratory evaluation revealed severe hypokalemia and hyperglycemia without ketoacidosis, although no diabetes mellitus was previously known. Computed tomography revealed a large tumor obliterating the left paranasal sinus and a left-sided adrenal mass. After cardiovascular stabilisation, a thorough hormonal assessment was performed revealing marked adrenocorticotropic hormone-dependent hypercortisolism. Due to the presence of a cardiac pacemaker magnetic resonance imaging of the hypophysis was not possible. [^68^Ga-DOTA]-TATE-Positron-Emission-Tomography was performed, showing somatostatin-receptor expression of the paranasal lesion but not of the adrenal lesion or the hypophysis. The paranasal tumor was resected and found to be an adrenocorticotropic hormone-producing paraganglioma of low-proliferative rate. Postoperatively the patient became normokaliaemic, normoglycemic and normotensive without further need for medication. Genetic testing showed no mutation of the succinatdehydrogenase subunit B- and D genes, thus excluding hereditary paragangliosis.

**Conclusion:**

Detection of the adrenocorticotropic hormone source in Cushing’s syndrome can prove extremely challenging, especially when commonly used imaging modalities are unavailable or inconclusive. The present case was further complicated by the simultaneous detection of two tumorous lesions of initially unclear biochemical behaviour. In such cases, novel diagnostic tools - such as somatostatin-receptor imaging - can prove useful in localising hormonally active neuroendocrine tissue. The clinical aspects of the case are discussed and relevant literature is reviewed.

## Background

Paragangliomas are extra-adrenal neuroendocrine tumors that arise from chromaffin cells in the sympathetic (localized in retroperitoneum and thorax) or parasympathetic (next to aortic arch, neck, and skull base) neural paraganglia [1]. Most paragangliomas are either asymptomatic or present as a painless mass and – although all contain neurosecretory granules - in only 1–3% of all cases secretion of hormones is abundant enough to be clinically significant. Although numerous reports of adrenocotricotropic hormone (ACTH)-producing adrenal phaeochromocytomas exist, ACTH-producing extraadrenal paragangliomas are extremely rare. A limited number of ACTH-expressing intraabdominal [1-5] or intrathoracic [6-11] paragangliomas can be found in literature, however, to our knowledge, only two cases of ACTH-expressing nasal paragangliomas have been reported [12,13]. Furthermore, there have been four reports of ectopic ACTH-secreting pituitary adenomas, three of them localised in the sphenoidal sinus [14-16] and one intracavernously [17] as well as seven reports of ACTH-secreting olfactory neuroblastomas [18-24]. We present a rare case of severe hypercortisolemia due to ectopic ACTH secretion by a nasal paraganglioma.

## Case presentation

### History

A 70-year-old Caucasian woman presented to the emergency department of our hospital with palpitations, facial oedema and hypertensive crisis. The patient reported fatigue, shortness of breath, coarseness of nose and throat and progressive oedema of the face over the past year. She declined weight loss, fever, chills, diarrhoea or further gastrointestinal complaints. Her family doctor had initially suspected an allergic reaction and had treated her with a course of prednisolone (40 mg/d) over two weeks with no improvement of symptoms. Three years ago she had been diagnosed with sick sinus syndrome and had received a cardiac pacemaker. Other than arterial hypertension, treated by ramipril, no further comorbidities were reported. She did not smoke, drink alcohol or have any known allergies. Family history was unremarkable.

### Clinical examination

Clinical examination revealed pitting oedema and redness of face, tachycardia and hypertension. Chest auscultation war normal, her abdomen was tender without signs of distention or peritonism. The neurological examination was unremarkable.

### Laboratory and electrocardiographic findings

Initial laboratory evaluation revealed severe hypokalaemia (K^+^: 1,7 mmol/L), hyperglycemia without ketoacidosis and mild leucocytosis. An electrocardiogram showed supraventricular tachycardia. After haemodynamic stabilisation, a thorough hormonal assessment was performed, which revealed markedly increased serum and urinary cortisole and increased plasma ACTH. Plasma and urine katecholamines and metanephrines as well as the aldosterone/renin ratio were within normal range (Table 1).

**Table 1:** Results of baseline hormone values and 24-hour urinary cortisole

**Parameter**	**Value**	**Reference range**
ACTH	273 pg/ml	[5–50]
Cortisole	74.4 μg/100 ml	[5–25]
DHEAS	3.1 ng/ml	[1.3–9.8]
Aldosterone	67.1 pg/ml	[10–160]
Plasma renin activity	1.7 ng/ml/h	[0.2–2]
Adrenaline	22 ng/l	[< 84]
Noradrenaline	104 ng/l	[< 420]
Dopamine	19 ng/l	[< 85]
Metanephrine	<5 ng/l	[< 90]
Normetanephrine	19 ng/l	[< 200]
24-hour urinary cortisol	6551.2 μg/24 h	[20–90]

### Imaging studies

**Figure 1:**
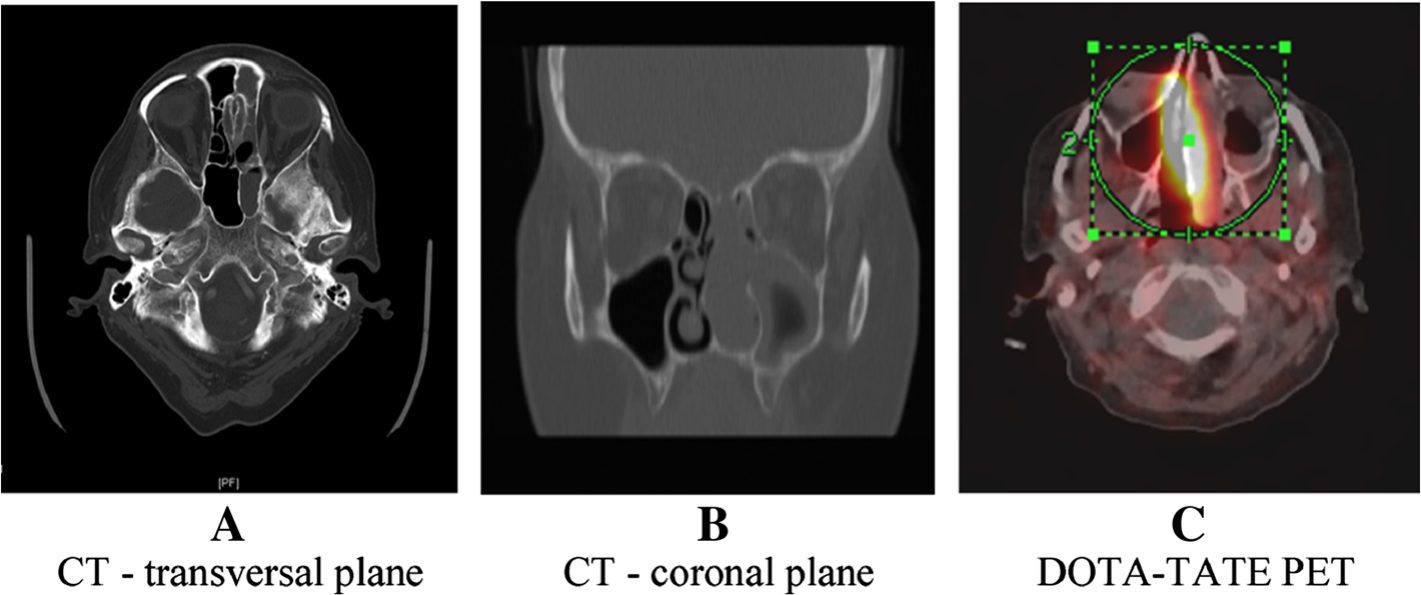
**Computed tomography and [68Ga-DOTA]-TATE-Positron-Emission-Tomography of an ACTH-producing tumor localised in the left paranasal sinus. A**: Computed tomography (**A**: coronal, **B**: transversal) and [^68^Ga-DOTA]-TATE-Positron-Emission-Tomography coregistered with computed tomography **(C)**, displaying a tumorous lesion completely obliterating the left paranasal sinus.

Due to initially suspected pneumonia (leucocytosis, shortness of breath), computed tomography of the chest was performed, which did not show pulmonary infiltrations. However a left adrenal mass of 1 cm diameter was detected in the lower computed-tomography (CT)-slices. Since no magnetic resonance imaging (MRI) was possible due to the presence of a cardiac pacemaker, pituitary imaging was performed by computed tomography. Brain-CT revealed no pathology of the sella region, however a large tumor obliterating the left paranasal sinus was detected (Figure 1A,B). We discussed about performing a high dose dexamethasone-suppression test to differentiate between orthotopic and ectopic ACTH secretion but decided against it, given the relatively poor predictive value of the test [25] and its low diagnostic consequence in the current setting (the adreanal and paranasal masses had to be worked up irrespective of test results). Furthermore, although the dexamethasone-suppression test is not contraindicated in diabetes, we did not want to risk a new aggravation of glucose homeostasis. At this point we decided to perform somatostatine receptor imaging since the level of suspicion for ectopic ACTH production was high and initially considered an Octreoscan. However, since we were already confronted with two tumorous lesions, we sought an imaging modality with higher resolution and, taking a similar, recently published case report into account [16], decided to perform a whole-body somatostatine-receptor Positron-Emission-Tomography (PET) scan with [^68^Ga-DOTA]-TATE. The scan revealed markedly elevated somatostatine-receptor expression of the paranasal tumour but not of the sellar region or the left adrenal mass (Figure 1C). Based on these results, total excision of the paranasal tumor with bone reconstruction was performed.

### Histological findings

**Figure 2:**
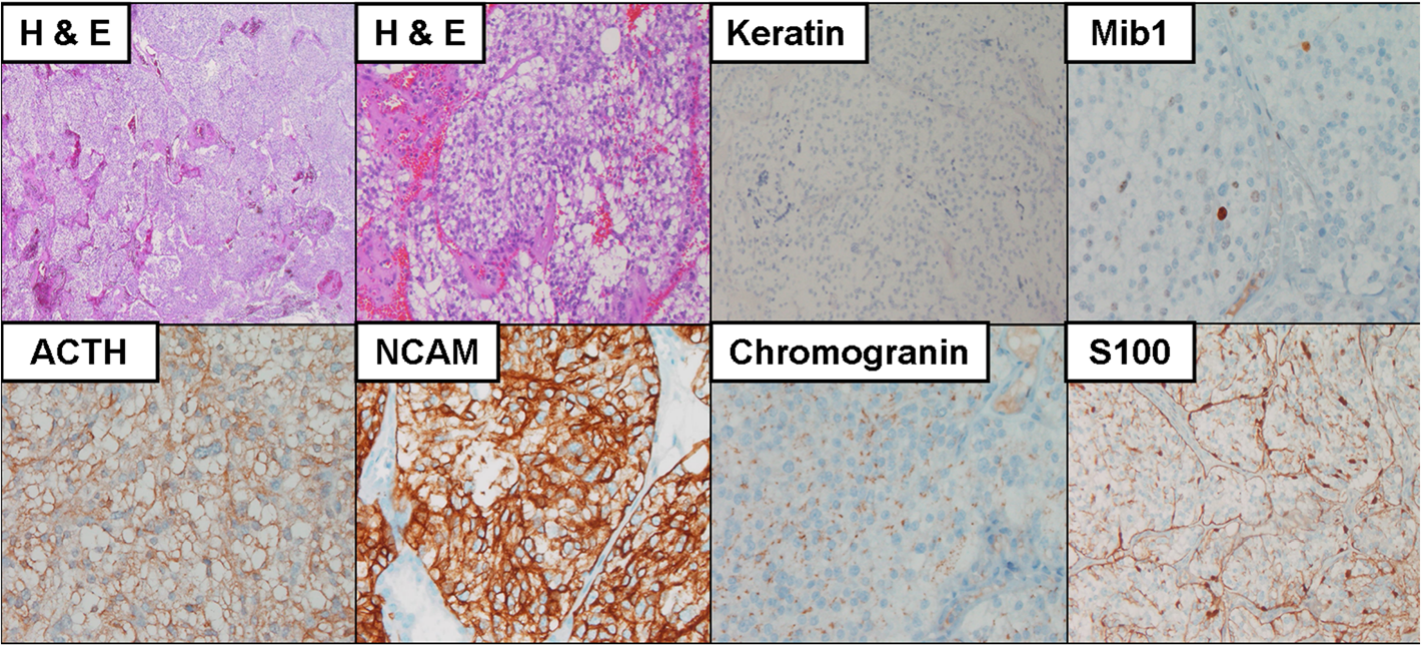
Hematoxylin and eosin (H&E) stain of paraffin embedded tumor tissue and immunohistochemistry with antibodies specific for keratin, S100, proliferation marker Mib1, adrenocotricotropic hormone (ACTH) and neuroendocrine markers NCAM and chromogranin.

Initial histopathological assessment revealed an ACTH-expressing neuroendocrine tumor with the differential diagnosis including ectopic pituitary adenoma, olfactory neuroblastoma and nasal paraganglioma. The final diagnosis of paraganglioma was confirmed by two independent pathologists, based on morphological findings, immunohistochemical profile and the low-proliferative activity of the tumor (Ki67<2%) (Figure 2).

### Treatment and postoperative course

The patient was initially treated by intravenous fluids, potassium and low-dose insulin. After haemodynamic stabilisation and localising studies, the tumor was resected by a transnasal/transsphenoidal approach followed by bone reconstruction. Postoperatively there was complete remission of hypertension, hypokalaemia, hyperglycemia and oedemas. Due to the chronic suppression of the corticotropic axis, adrenal insufficiency ensued after removal of the ectopic ACTH source, rendering transient hydrocortisone substitution necessary. The adrenal mass was classified as incidentaloma, since all hormonal activity seized after removal of the paranasal tumor and no growth tendency was observed in radiologic follow up.

### Genetic testing

About 75% of paragangliomas are sporadic; the remaining 25% are hereditary and have an increased likelihood of being multiple and of developing at an earlier age. After obtaining written informed consent, we performed genetic testing which revealed no mutation of the succinate dehydrogenase subunit B (*SDHB*) and D (*SDHD*) genes thus excluding hereditary paragangliosis. Since no other features of multiple endocrine neoplasia (MEN) syndrome were apparent, we characterised the tumor as sporadic and decided against testing for mutations of the *RET*-protooncogene.

## Conclusions

Detection of the ACTH source in Cushing’s syndrome can prove extremely challenging, especially when commonly used imaging modalities are unavailable or inconclusive. In such settings, novel diagnostic modalities, such as somatostatin-receptor imaging, can prove useful in localising hormonally active neuroendocrine tissue. In the present case we were confronted by a very rare ACTH-producing nasal paraganglioma. The diagnostic procedure was complicated by the simultaneous discovery of two tumorous lesions and the unavailability of MRI due to the presence of a cardiac pacemaker. The ectopic ACTH-source was eventually localised by [^68^Ga-DOTA]-TATE-PET and was surgically removed, which led to complete remission of the hypercortisolaemic syndrome.

## Consent

Written informed consent was obtained from the patient for publication of this Case Report and any accompanying images. A copy of the written consent is available for review by the Editor-in-Chief of this journal.

## Abbreviations

ACTH: Adrenocotricotropic hormone; CT: Computed tomography; MRI: Magnetic resonance imaging; PET: Positron emission tomography; SDHB: Succinate dehydrogenase subunit B; SDHD: Succinate dehydrogenase subunit D; MEN: Multiple endocrine neoplasia.

## Competing interests

The authors declared that they have no competing interests.

## Authors’ contributions

TT drafted the manuscript and was involved in patient management. SZ aided in drafting the manuscript and was involved in patient management. CT, EJ and MPM supervised patient management and critically read and revised the manuscript. All authors read and approved the final manuscript.

## Authors’ information

TT and SZ are junior physicians training in endocrinology. CT and EJ are senior physicians specialising in endocrinology and gastroenterology. MPM is the chief of the Department of Gastroenterology, Hepatology and Endocrinology of the Hannover Medical School.
